# Role of NFE2L1 in the Regulation of Proteostasis: Implications for Aging and Neurodegenerative Diseases

**DOI:** 10.3390/biology12091169

**Published:** 2023-08-25

**Authors:** Aswathy Chandran, Haley Jane Oliver, Jean-Christophe Rochet

**Affiliations:** 1 Department of Medicinal Chemistry and Molecular Pharmacology, Purdue University, West Lafayette, IN 47907, USA; 2 Purdue Institute for Integrative Neuroscience, Purdue University, West Lafayette, IN 47907, USA

**Keywords:** NFE2L1, NRF2, proteasome, antioxidant response element, neurodegenerative diseases, aging

## Abstract

**Simple Summary:**

As we age or develop certain brain diseases, our body’s ability to maintain a balanced protein system, termed “proteostasis”, is disturbed. Proteostasis is essential for keeping proteins at the correct levels and functioning properly, as well as avoiding a buildup of proteins that form aggregates involved in neurodegenerative diseases, like Alzheimer’s disease and Parkinson’s disease. One of the major pathways used by our cells to eliminate such proteins is the “ubiquitin proteasome system” (UPS). In this review, we discuss how problems with the UPS are linked to aging and neurodegenerative diseases. We also explore the idea that increasing the activity of NFE2L1, a protein that increases expression of UPS components, could be a strategy to enhance proteasome function and reduce the impact of neurodegenerative diseases.

**Abstract:**

A hallmark of aging and neurodegenerative diseases is a disruption of proteome homeostasis (“proteostasis”) that is caused to a considerable extent by a decrease in the efficiency of protein degradation systems. The ubiquitin proteasome system (UPS) is the major cellular pathway involved in the clearance of small, short-lived proteins, including amyloidogenic proteins that form aggregates in neurodegenerative diseases. Age-dependent decreases in proteasome subunit expression coupled with the inhibition of proteasome function by aggregated UPS substrates result in a feedforward loop that accelerates disease progression. Nuclear factor erythroid 2- like 1 (NFE2L1) is a transcription factor primarily responsible for the proteasome inhibitor-induced “bounce-back effect” regulating the expression of proteasome subunits. NFE2L1 is localized to the endoplasmic reticulum (ER), where it is rapidly degraded under basal conditions by the ER-associated degradation (ERAD) pathway. Under conditions leading to proteasome impairment, NFE2L1 is cleaved and transported to the nucleus, where it binds to antioxidant response elements (AREs) in the promoter region of proteasome subunit genes, thereby stimulating their transcription. In this review, we summarize the role of UPS impairment in aging and neurodegenerative disease etiology and consider the potential benefit of enhancing NFE2L1 function as a strategy to upregulate proteasome function and alleviate pathology in neurodegenerative diseases.

## 1. Introduction: The Ubiquitin Proteasome System

Cellular homeostasis depends critically on the strict regulation of intracellular protein levels. A fine balance exists between protein synthesis and degradation, two processes that are both interdependent and tightly coordinated through the regulation of multiple upstream and downstream pathways [1]. Continuous protein degradation is vital not only to avoid the accumulation of unneeded or misfolded proteins, but also to maintain free amino acid pools for protein synthesis [2]. Dietary amino acids constitute only about 20% of the amino acids required to sustain normal protein synthesis in humans, whereas the remaining 80% are obtained by recycling existing proteins [3].

One of the most important protein degradation pathways is the ubiquitin proteasome system (UPS). In carrying out the turnover of a broad array of proteins, the UPS regulates a variety of cellular functions such as cell division, apoptosis, and cell signaling [4,5]. Protein degradation by the UPS involves two steps: (i) conjugation of ubiquitin (Ub), a 76-residue polypeptide that serves as a signal for protein degradation, to a target protein; and (ii) degradation of the ubiquitinated protein by the proteasome. The 26S proteasome is a 2.5 MDa protein complex consisting of a 20S core particle (CP) bound to one or two 19S regulatory particles (RPs) [6]. The 20S CP consists of four rings of heptamers and serves as the site of proteolytic degradation within the 26S proteasome. The two outer rings are comprised of alpha subunits (α1–α7) that control access to the core chamber. Conversely, the inner rings consist of beta subunits (β1–β7) that form the catalytic region, with the β1, β2, and β5 subunits having caspase-, trypsin-, and chymotrypsin-like activities, respectively. The 19S RP complex functions to control recognition, deubiquitination, unfolding, and translocation of substrates into the 20S CP. It consists of two parts: the base and the lid. The base is formed from six AAA^+^ ATPases, termed Rpt1–6, and four non-ATPase subunits, termed Rpn1, Rpn2, Rpn10, and Rpn13. The base sits in direct contact with the outer alpha rings of the 20S CP. The lid consists of nine non-ATPase subunits: Rpn3, Rpn5–9, Rpn11, Rpn12, and Sem1.

Ubiquitination, the primary biochemical modification targeting cellular proteins for degradation [7], involves the covalent addition of Ub to a target protein through a series of enzymatic reactions. Ub is first activated by an E1 Ub-activating enzyme in an ATP-dependent reaction, and the activated Ub moiety is then transferred to an E2 conjugating enzyme. Lastly, an E3 ubiquitin ligase catalyzes the transfer of the Ub molecule from the E2 enzyme to lysine residues on the target protein. This process is repeated many times to generate a polyubiquitin (poly-Ub) chain. Poly-Ub chains produced by repeatedly attaching a Ub molecule to residue K48 on the preceding Ub molecule form the major signal for proteasomal degradation. However, mammalian proteasomes can also degrade singly and multiply monoubiquitinated substrates, as well as proteins with non-K48 linked polyubiquitin chains [8]. The 26S proteasome-mediated degradation of a ubiquitinated protein target involves an initial Ub recognition step that is primarily mediated by subunits Rpn10 and Rpn13 of the 19S RP [8,9], followed by deubiquitination of the substrate by Rpn11 or one of two other proteasome-associated deubiquitinating enzymes (DUBs), UCH37 and USP14. The target protein is then unfolded in an ATP-dependent manner by the Rpt subunits in the base of the 19S RP. A study performed using denatured citrate synthase revealed that the base also possesses chaperone activity that serves to refold protein substrates in an ATP-dependent manner [10].

Proteasomal degradation is also induced by the presence of a disordered (unstructured) region in the target protein [11,12]. Disordered regions (either unmodified or ubiquitinated) can serve as initiation sites for proteasomal degradation and enhance the efficiency of proteolysis significantly [13]. The 20S CP can exist as an independent entity (i.e., lacking a 19S RP) in cells [14], and in this form it can degrade short peptides and unfolded proteins in an ATP- and ubiquitin-independent manner [15]. Apart from the standard 26S proteasome complex, there are alternate versions of the proteasome that can be formed in response to specific physiological stimuli. Early experiments with IFNγ led to the discovery of a new proteasome complex in which the catalytic subunits β1, β2, and β5 are substituted by β1i, β2i, and β5i [16,17]. This complex, termed the immunoproteasome, has been found to play an important role in generating peptides for MHC class I antigen presentation. Additionally, in epithelial cells of the thymus, a novel catalytic subunit β5t is incorporated into the proteasome along with β1i and β2i to form a complex called the thymoproteasome, which has been found to play a role in T-cell development [17]. The 20S CP can also be capped by PA200 and PA28, proteasome activator complexes that function by constitutively opening the gate controlling access to the 20S CP [18,19]. Unlike the 19S RP, these complexes are unable to recognize ubiquitin and, therefore, aid in the degradation of nonubiquitinated proteins only. The PA28 complex is induced by IFNγ and plays a role in processing peptides for antigen presentation [20].

## 2. Role of the UPS in Aging and Neurodegenerative Diseases

A disruption of proteome homeostasis (“proteostasis”) is one of the hallmarks of brain aging [21]. Age-associated impairment of the UPS, as well as other proteostasis mechanisms including the autophagy-lysosome pathway (ALP) and the heat-shock response (both described in more detail below), can lead to a buildup of toxic protein aggregates [22]. Post-mitotic cells such as neurons are particularly vulnerable to altered proteostasis due to their inability to clear damaged and oxidized proteins through cell division. UPS-mediated protein degradation may be affected by decreased expression levels of proteasome subunits, improper assembly of functional proteasomal complexes, or impairment of proteasomal activity by protein aggregates. As one example, an age-related reduction in the expression levels of certain proteasome subunits has been observed in mouse skeletal muscle [23]. In addition, late-passage human fibroblasts showed a decline (relative to early-passage cells) in the expression of various proteasome subunits that correlated with an accumulation of ubiquitinated and oxidized proteins and an upregulation of senescence markers [24]. Similar results were obtained when comparing human fibroblasts from young versus old donors. Moreover, proteasomal chymotryptic activity was found to decrease with age in rat brain [25], and proteasome subunit expression levels and proteasomal activity were reduced in the spinal cords of aged rats [26].

A comparison of proteomic and transcriptomic signatures in the brains of young, adult, and old killifish (*Nothobranchius furzeri*) revealed a significant lack of correlation between overall transcript and protein levels with increasing age [27]. The authors also noted an age-dependent disruption of the subunit stoichiometry and assembly of various protein complexes such as the ribosome and mitochondrial electron transport chain. These phenotypes may result from an age-dependent decrease in proteasome activity. Consistent with this idea, the ratio of free 20S CP to capped 26S proteasome was increased in adult fish. Moreover, proteasome subunit expression levels and the proteolytic activities of the 26S proteasome were found to decrease progressively with age. Collectively, these data establish the importance of proteasomal activity in maintaining the integrity of various protein complexes, the dysfunction of which could contribute to aging via the perturbation of multiple cellular pathways.

In addition to contributing to physiological aging, impairment of the UPS plays a major role in the pathophysiology of multiple neurodegenerative diseases (NDs) including Parkinson’s disease (PD), Alzheimer’s disease (AD), and amyotrophic lateral sclerosis (ALS). Mutations in several genes that are a part of the UPS pathway are implicated in familial NDs. Parkin, an E3 ubiquitin ligase associated with autosomal recessive PD [28], modulates the turnover of proteins involved in cellular processes such as synaptic function and mitophagy. Another UPS effector associated with familial ND, the deubiquitinating enzyme ubiquitin carboxy-terminal hydrolase L1 (UCHL1), is responsible for removing ubiquitin tags from UPS substrates, thereby maintaining the free ubiquitin pool in neurons [29]. Several mutations in UCHL1, including I93M and E7A, are associated with familial PD [30] and an early-onset, progressive neurodegenerative disorder involving cerebellar ataxia and upper motor neuron dysfunction [31], respectively. Wild-type (WT) UCHL1 has also been implicated in idiopathic PD and AD and is enriched in the characteristic intraneuronal inclusions associated with these two disorders, Lewy bodies and neurofibrillary tangles (NFTs), respectively. Multiple UPS-related genes are also implicated in ALS pathogenesis. Mutations in ubiquilin-2, a ubiquitin-like protein, can cause familial X-linked ALS/dementia [32]. Ubiquitin-like proteins act as adapters to facilitate the binding of polyubiquitinated substrates to the proteasome, and the expression of mutant ubiquilin-2 can cause impaired proteasomal degradation and the accumulation of ubiquitinated substrates [32,33]. Valosin-containing protein (VCP)/p97 is an AAA^+^ ATPase that aids in degrading ubiquitinated proteins associated with different cellular organelles, including the endoplasmic reticulum (ER) and mitochondria, by unfolding and facilitating the release of the target from protein complexes [34]. Mutations in VCP have been identified in families with autosomal dominant forms of ALS and cause motor neuron loss and aggregation of TDP-43 (transactive response DNA binding protein 43 kDa) [35].

NDs are also characterized by defects in mitochondrial respiration and a redox imbalance that can in turn lead to altered UPS function. Reactive oxygen species (ROS) and reactive nitrogen species (RNS) are free radicals with unpaired electrons typically produced as a result of normal cellular metabolism, and they tend to accumulate with aging and in response to inflammation or UV radiation [36]. ROS accumulation leads to the oxidation of cellular components including DNA, lipids, and proteins, eventually resulting in cell death. Oxidized proteins have been shown to be preferentially degraded by the 20S proteasome in an ATP- and ubiquitin-independent manner.

The proteasome is itself the target of numerous oxidative post-translational modifications, including S-glutathionylation, HNE-induced adduct formation, protein carbonyl formation, and glycoxidation, all of which affect proteasome function to different extents [37]. The physiological function of the free 20S CP is prevalent under conditions of oxidative stress [38,39], which favor dissociation of the 20S CP from the 19S RP [40]. The 20S CP is relatively resistant to an oxidative environment, whereas the 26S proteasome is more susceptible to oxidative damage, losing its proteolytic activity in the presence of low amounts of ROS that have no impact on the 20S CP [41].

## 3. Degradation of Amyloidogenic Proteins: A Role for the UPS vs. ALP

In addition to its role in proteostasis, the UPS is responsible for clearing aggregation-prone proteins involved in NDs. Any impairment of proteasome function could thus cause protein aggregation and adversely affect the cell via a toxic gain of function. Most NDs including PD, AD, ALS, and Huntington’s disease (HD) have a unifying dominant feature—namely, the formation and spreading of protein aggregates. The structures and conformations of these protein aggregates vary significantly depending on local cellular conditions [42,43], and extensive research has been carried out to identify the neurotoxic species primarily responsible for disease pathogenesis. Most protein aggregation events follow a sigmoidal growth curve with a lag phase involving nucleation of the monomeric protein to form higher order oligomers or protofibrils, which then assemble to form longer fibrillar aggregates in an accelerated growth phase [44]. Apart from these on-pathway oligomers, various off-pathway oligomers that fail to convert to mature fibrils have also been reported [45,46]. There is no clear consensus about the nature of the proteinaceous species responsible for neuron death in neurodegenerative disease: although most studies point to prefibrillar oligomers [42,47,48,49,50], some findings suggest that mature fibrils also contribute to toxicity [51,52,53,54,55]. Although the major constituent protein and the cell types implicated in these disorders differ, defects in protein clearance pathways have been implicated in all these diseases. The role of the UPS in the etiology of NDs has been discussed extensively in recent years [56,57,58]. Most NDs are caused by the misfolding and aggregation of amyloidogenic proteins (i.e., proteins with a high propensity to form β-sheet-rich amyloid or amyloid-like fibrils), a process that is likely promoted by age- or disease-related UPS impairment given that these proteins tend to be UPS substrates [22,56]. 

In addition to the UPS, the autophagy-lysosome pathway (ALP) plays a major role in cellular protein clearance. The term “ALP” includes multiple processes—namely, macroautophagy, microautophagy, and chaperone-mediated autophagy. Macroautophagy (referred to as autophagy hereafter) involves the encapsulation of cellular cargo in membrane-bound vesicles called autophagosomes, which then fuse to lysosomes, resulting in degradation of the intravesicular contents by lysosomal enzymes [59]. Both the UPS and autophagy are involved in the clearance of amyloidogenic proteins from cells, and the degree to which each is involved in the degradation of a given protein substrate depends on the protein’s aggregation state, conformation, and ubiquitination status [60,61]. Evidence from studies in cell-free systems suggests that α-synuclein (aSyn), a presynaptic protein enriched in fibrillar form in Lewy bodies in the brains of PD patients, can be degraded in the unmodified or monoubiquitinated state by the 20S or 26S proteasome, respectively [62,63,64]; However, other studies have revealed that aSyn ubiquitination is essential for proteasomal degradation in cell culture. Rott et al. [65] found that monoubiquitination of aSyn by SIAH, an E3 ubiquitin ligase, facilitated preferential proteasomal degradation with minimal contribution from the autophagy pathway, whereas deubiquitination by the enzyme USP9X facilitated aSyn accumulation. Surprisingly, the authors also found that the deubiquitinated fraction of aSyn was preferentially cleared via the ALP rather than the UPS, suggesting that the ubiquitination status of aSyn determines its degradation pathway. Additional findings suggest that aSyn variants with different fibrillization propensities are degraded via different mechanisms. For example, WT aSyn and the familial PD mutant A30P were found to be eliminated from cultured rat pheochromocytoma PC12 cells by the UPS, whereas the more fibrillogenic mutant A53T was degraded by both the UPS and autophagy [66]. Although monomeric aSyn is an established target of the proteasome in cell-free systems, the nature of the aSyn species degraded by the UPS in an intracellular environment is not fully established. In one study, Emmanouilidou et al. [67] found that oligomeric (but not monomeric) aSyn accumulated in A53T-expressing PC12 cells treated with proteasome inhibitors, implying that the UPS preferentially targets aSyn oligomers in cells. Additional findings suggest that the proteasome can disaggregate nonubiquitinated aSyn fibrils, although this phenomenon seems to depend on the protein unfolding role of the 19S RP rather than proteasomal proteolytic activity per se [68]. A study of the degradation pathways involved in eliminating distinct aSyn conformers revealed that PFF-induced aSyn aggregates, visualized using an aggregate-specific antibody that recognizes aSyn, were predominantly cleared by the lysosome as opposed to the UPS [69]. However, a variant of aSyn phosphorylated on residue S129, apparently existing in oligomeric and monomeric forms, was found to be more abundant in cells treated with the proteasome inhibitor epoxomicin. Collectively, these studies point to a defined role of the UPS in degrading monomeric as well as phosphorylated, oligomeric aSyn species.

Other amyloidogenic proteins implicated in NDs are also targets of the UPS. The microtubule-associated protein tau, hyperphosphorylated forms of which are converted to NFTs in the brains of AD patients, undergoes ubiquitin-independent or -dependent degradation by the 20S or 26S proteasome, respectively [70,71]. TDP-43 and mutant Cu/Zn-superoxide dismutase (SOD1), both of which are involved in the pathogenesis of ALS, are also cleared by the UPS as well as by autophagy [72,73].

## 4. Inhibition of the UPS by Amyloidogenic Proteins

Impairment of the UPS in NDs has been widely attributed to the presence of high-molecular-weight protein aggregates that can bind to the proteasome, thereby blocking substrate entry and processing [67,74,75]. Amyloidogenic proteins can adopt different multimeric states, a subset of which appear to be responsible for proteasome inhibition. Recombinant, oligomeric forms of aSyn, huntingtin, and the amyloid-β peptide associated with AD, all of which are characterized by the presence of a unique epitope referred to as the A11 epitope, were found to inhibit the proteolytic activity of the proteasome, thereby causing the accumulation of UPS substrates, whereas monomeric and large insoluble fibrillar forms of these proteins had no inhibitory effect [76]. It was inferred from subsequent experiments that oligomers with the A11 epitope destabilize the gating mechanism of the 20S and 26S proteasome, thereby impairing substrate entry. This inhibitory effect was suggested to involve an allosteric mechanism because the A11^+^ oligomers are too large to enter the translocation channel and clog the active sites of the proteasome. Furthermore, when incubated with a constitutively active, “open gate” form of the 20S proteasome containing α3 subunit with an N-terminal deletion, the oligomers failed to inhibit proteolytic activity, consistent with a noncompetitive inhibition mechanism. Other studies revealed that low-molecular-weight aggregates of recombinant tau interfered with ATP hydrolysis by the 19S subunit [77], suggesting that amyloidogenic proteins can inhibit UPS function by disrupting the proteasome’s ability to use ATP as a source of energy in addition to its proteolytic activity.

UPS inhibition by amyloidogenic proteins has also been demonstrated in cellular and animal models, confirming that this phenomenon is physiologically relevant. In one study, UPS proteolytic activity was found to be reduced to a greater extent in PC12 cells overexpressing A30P-aSyn versus the WT protein [78]. Moreover, mice expressing A53T-aSyn in the substantia nigra were shown to have reduced proteasome activity, leading to the accumulation of UPS substrates and the formation of polyubiquitinated inclusions [79]. A reduction in proteasome activity was also observed in transgenic mice expressing the P301L tau mutant linked with familial tauopathy disorders [77]. Transgenic mice expressing mutant SOD1 also showed evidence of proteasomal dysfunction leading to a buildup of UPS substrates, although this phenomenon was attributed to a decrease in levels of both the 19S RP and 20S CP [80,81].

Together, these findings suggest that UPS impairment inNDs can arise from mutations in genes encoding UPS effectors, as well as from inhibition or downregulation of the proteasome elicited by protein oligomers [56]. Because UPS dysfunction resulting from these different mechanisms presumably favors the aggregation and neurotoxicity of amyloidogenic proteins involved in NDs, extensive research efforts have been focused on developing therapies aimed at enhancing UPS-mediated protein clearance in the brains of patients with these disorders.

## 5. Upregulation of the UPS as a Therapeutic Strategy for NDs (I): Stimulation of Proteasome Activity

Stimulation of UPS-mediated protein degradation, a potential therapeutic strategy for NDs [82], can be achieved by increasing the activity of the proteasome via (i) regulation of proteasome subunit phosphorylation or (ii) modulation of substrate ubiquitination (discussed below). Various cellular kinases can phosphorylate the proteasome in response to physiological stimuli [83], including protein kinase A (PKA), a kinase activated by cyclic adenosine monophosphate (cAMP). PKA-mediated Rpn6 phosphorylation was found to induce an increase in proteasome activity and stimulate the degradation of aggregation-prone proteins such as tau, TDP-43, and FUS (fused in sarcoma), an RNA binding protein involved in ALS and frontotemporal dementia (FTD) [84]. Transgenic mice expressing the FTD-linked tau mutant P301L showed a decrease in cognitive deficits and reduced levels of insoluble and hyperphosphorylated tau upon administration of rolipram, a treatment that led to an increase in proteasome activity in the brain [77]. Another kinase implicated in the regulation of proteasome activity is p38 MAPK, previously reported to inhibit the proteasome via Rpn2 phosphorylation under conditions of osmotic stress [85]. Consistent with such an inhibitory effect, pharmacological or siRNA-mediated downregulation of the p38 MAPK pathway was found to stimulate proteasome activity and decrease aSyn aggregation in a mammalian cell line [86].

UPS-mediated protein clearance can also be stimulated with treatments that favor the ubiquitination of proteasome substrates. One approach involves inhibiting DUBs that slow substrate turnover by catalyzing polyubiquitin chain “trimming” prior to the initiation of proteolytic degradation. For example, inhibition of the DUB USP14 with the small molecule IU1 was shown to enhance proteasome activity as well as the clearance of tau and TDP-43 in mouse embryonic fibroblasts [87]. Another approach is to use heterobifunctional peptides or small molecules known as “proteolysis targeting chimeras” (PROTACs) to recruit proteasome substrates to an E3 ubiquitin ligase, thereby promoting target-specific ubiquitination and degradation. PROTAC-mediated turnover is actively being explored as a therapeutic strategy to accelerate the clearance of amyloidogenic proteins such as tau and aSyn, thus preventing their aggregation [88,89,90]. 

## 6. Upregulation of the UPS as a Therapeutic Strategy for NDs (II): Induction of Proteasome Subunit Expression

**Figure 1:**
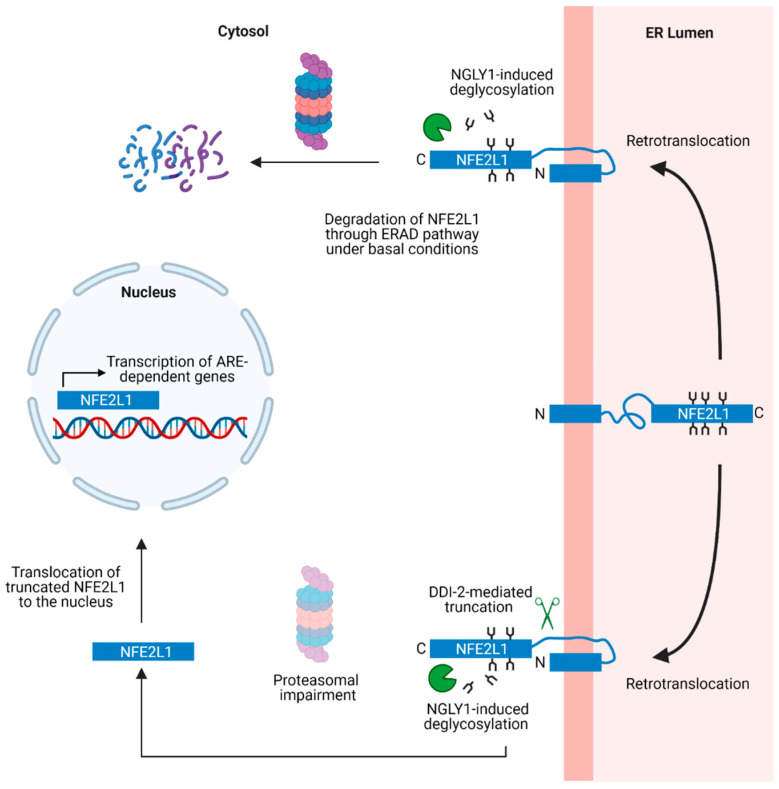
Mechanism of NFE2L1 activation. (**Top**) Under basal conditions, ER-associated NFE2L1 is retrotranslocated to the cytosolic side and undergoes deglycosylation by the N-glycanase NGLY1, followed by ERAD-dependent proteasomal degradation. (**Bottom**) Under conditions of proteasome impairment, NFE2L1 is deglycosylated by NGLY1 and N-terminally cleaved by the protease DDI-2. The cleaved fragment is translocated to the nucleus, where it modulates the transcription of ARE-dependent genes. Figure created with BioRender.com, accessed on 19 June 2023.

UPS-mediated protein degradation can also be stimulated by inducing proteasome subunit expression and promoting the assembly of functional proteasome complexes. Proteasome inhibition leads to the activation of a stress response pathway that results in the global upregulation of all proteasome subunits [91]. This regulatory mechanism, referred to as the “bounce-back” response, is observed in all eukaryotic cells and is responsible for the emergence of chemotherapeutic resistance following treatment with proteasome inhibitors. In mammalian cells subjected to proteasome impairment, proteasome subunit expression is activated by the transcription factor nuclear factor erythroid 2-like 1 (NFE2L1), also referred to as Nrf1 (not to be confused with nuclear respiratory factor-1, also referred to as Nrf1 in the literature). NFE2L1/Nrf1 belongs to the cap ‘n’ collar-basic leucine zipper (CNC-bZIP) family of transcription factors, which includes p45 NF-E2, NFE2L2/Nrf2, Nrf3, Bach1, and Bach2 in humans, CncC in *Drosophila melanogaster*, and SKN-1 (an ortholog of both NFE2L1 and Nrf2) in *Caenorhabditis elegans* [92]. NFE2L1 is an ER-resident protein that undergoes p97-mediated retrotranslocation to the cytosol under basal conditions, leading to its degradation by the ERAD pathway [93] (Figure 1). In contrast, when the proteasome is inhibited, NFE2L1 is no longer degraded, but, rather, undergoes deglycosylation followed by N-terminal truncation at residue 104. In turn, truncated NFE2L1 translocates into the nucleus, where it interacts with small Maf proteins to form the active transcription factor. Although the cellular machinery responsible for NFE2L1 truncation was initially assumed to be the proteasome itself [94], subsequent research revealed that human NFE2L1 and its *C. elegans* ortholog SKN-1 are cleaved in the N-terminal region by the aspartyl protease DNA damage inducible 1 homolog 2 (DDI2) and DDI-1, respectively [95,96]. 

NFE2L1 stimulates the expression of proteasome subunit genes by binding to antioxidant response elements (AREs) in the promoter region [97]. Data from early studies suggested that the related, ARE-binding transcription factor NFE2L2 (better known as Nrf2) was the key transcription factor responsible for mediating the expression of proteasome subunit genes [98,99,100]; however, subsequent research revealed that NFE2L1 could upregulate these genes to a much greater degree than Nrf2 in mammalian cell lines [97,101]. Although both NFE2L1 and Nrf2 accumulate and translocate into the nucleus under conditions of proteasome inhibition, NFE2L1 has a higher affinity for the promoter region of proteasome subunit genes compared to Nrf2 based on data obtained via ChIP analysis [101].

Although it is clear that NFE2L1 plays an important role in regulating proteasome subunit expression in response to proteasome impairment, its role in regulating basal proteasome subunit expression is less established. Data from two studies revealed that NFE2L1 knockdown had no effect on proteasome subunit expression in mammalian cells cultured in the absence of proteasome inhibitors [97,101]. However, knockout of DDI2, the protease necessary for N-terminal truncation of NFE2L1, in the human colon cancer cell line HCT116 led to a decrease in proteasome activity, suggesting that NFE2L1 may be necessary for the basal expression of proteasome subunits [95]. Moreover, reduced basal proteasome subunit expression was observed in a conditional knockout mouse depleted of NFE2L1 in the liver [102]. Additionally, *C. elegans* strains lacking SKN-1 or PNG-1 (an enzyme necessary for SKN-1 deglycosylation) show evidence of reduced transcriptional activation of proteasome subunit genes as well as an accumulation of UPS substrates [103]. 

Other transcription factors that have been reported to affect basal proteasome subunit expression include Rpn4 in yeast [104], as well as nuclear transcription factor Y (NF-Y) [105], forkhead box protein O4 (FOXO4) [106,107], and signal transducer and activator of transcription 3 (STAT3) [108] in mammalian cells. 

## 7. mTORC1-Mediated Control of Proteasome Subunit Expression and Assembly: NFE2L1-Dependent and -Independent Mechanisms

Evidence suggests that NFE2L1 plays a role in the regulation of proteasome activity by autophagy. A study published by Zhang and colleagues [109,110] demonstrated that increased mTORC1 signaling caused by deletion of the negative regulator TSC2, a cellular perturbation expected to reduce autophagic flux, results in NFE2L1-dependent proteasome upregulation under conditions of serum starvation. SREBP1 (Sterol Regulatory Element Binding Protein 1), an ER-resident transcription factor downstream of mTORC1, can bind to regulatory regions of the NFE2L1 gene and induce NFE2L1 expression. Another group reported that activation of the mTORC1 pathway in embryonic fibroblasts from genetically modified mice with reduced mitochondrial complex I function results in the specific upregulation of the regulatory particle assembly chaperones (RACs) and the 19S subunit Rpn6, in turn leading to increased proteasome assembly and activity [111]. However, it is unclear if this effect is mediated by NFE2L1. This evidence that mTORC1 signaling can lead to proteasome activation contradicts data reported by other groups suggesting an opposite relationship between the UPS and the mTORC1 pathway [112,113]. In one such study performed by Rousseau et al. [112], rapamycin-mediated TORC1 inhibition in yeast and mammalian cells was found to cause a rapid yet transient increase in RAC and proteasome subunit expression. One possible reason for this discrepancy is that different experimental designs were used in the two studies. Acute inhibition of mTORC1 as carried out by Rousseau et al. [112] would be expected to lead to the upregulation of both autophagy and UPS-mediated protein degradation to clear any accumulated proteins and maintain the amino acid pool in cells, and the subsequent decline in UPS expression could potentially be explained by degradation of the proteasome by autophagy (proteaphagy) [6,114]. In contrast, long-term activation of the mTORC1 pathway caused by a deletion of TSC2, combined with serum starvation, as described by Zhang et al. [109], may lead to a different, NFE2L1-mediated adaptive response resulting in an overall upregulation of UPS function. The rapamycin-mediated increase in proteasome subunit expression observed by Rousseau et al. [112] was found to be dependent on the transcription factor Rpn4 in yeast, but whether NFE2L1 plays a role in this phenomenon in mammalian cells has not been determined. Although another group showed that inhibition of the TORC1 pathway leads to an increase in SKN-1 expression in *C. elegans* [115], levels of proteasome subunit expression or UPS activity were not measured in this study.

## 8. ER Stress-Induced NFE2L1 Activation

ER stress results from the accumulation of misfolded polypeptides and alterations of calcium homeostasis within the ER [116,117]. Perturbations of the ER or mitochondrial redox balance can also lead to changes in intracellular calcium flux that in turn exacerbate ER stress phenotypes [118]. In turn, these perturbations lead to the activation of various stress response pathways, collectively termed the unfolded protein response (UPR), that (i) induce an increase in chaperone expression to stimulate protein folding and the degradation of unwanted proteins; and (ii) temporarily inhibit translation to prevent a buildup of new proteins. The UPR is mediated by three major ER-resident signaling proteins—IRE1, PERK, and ATF6—that function in a coordinated manner to restore ER homeostasis. SKN-1 has been shown to play a role in the UPR in *C. elegans* by participating in a feedforward loop to mitigate ER stress [119]. Treatment of *C. elegans* with the ER stress inducer tunicamycin results in an SKN-1-dependent upregulation of multiple genes involved in the UPR, including IRE-1 and XBP-1. In turn, SKN-1 activation in response to ER stress is regulated by the UPR signaling proteins ATF6 and XBP-1. NFE2L1 was also shown to upregulate transcription of the ATF6 gene in mouse embryonic fibroblasts, and mice with a liver-specific NFE2L1 conditional knockout were found to have reduced basal expression levels of ATF6 and other ERAD factors [102]. Other studies revealed that NFE2L1 is a direct transcriptional activator of the ERAD pathway effectors Hrd1, an ER-resident E3 ubiquitin ligase [120], and Herpud1, a protein involved in retrotranslocation of substrates across the ER membrane for proteasomal degradation [121]. Collectively, these observations suggest that NFE2L1 induces the expression of proteins involved in the UPR in mammalian cells and in *C. elegans*. In contrast, however, serum-starved MCF10A cells (a human epithelial cell line) treated with chemical ER stress inducers such as tunicamycin and thapsigargin showed no increase in NFE2L1 levels [109], suggesting that further studies are needed to elucidate this pathway in different biological models.

**Figure 2:**
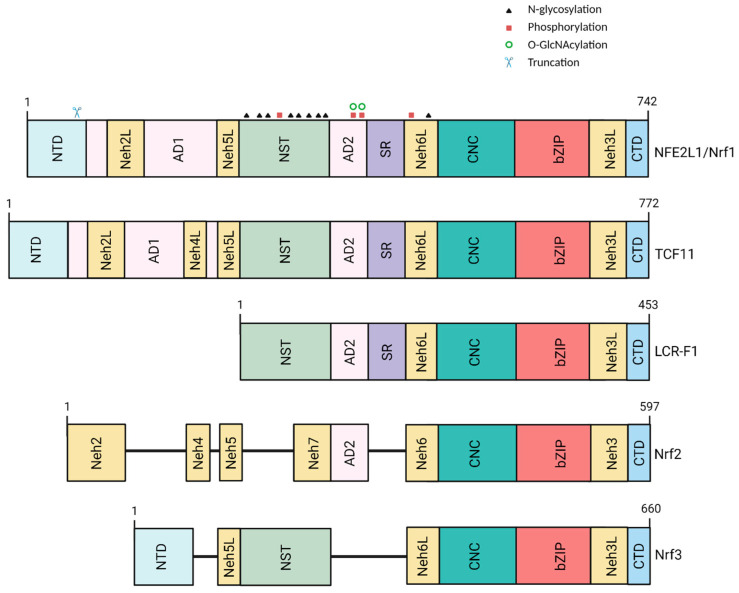
Schematic showing the domain architectures of three NFE2L1 isoforms (NFE2L1/Nrf1, TCF11, LCR-F1), Nrf2, and Nrf3. The domains are as follows: N-terminal domain (NTD); Nrf2-ECH Homology Like (Neh2L, Neh5L, Neh6L, Neh3L) domains; Acidic Domain 1 (AD1); Asparagine/Serine/Threonine (NST) domain; Acidic Domain 2 (AD2); Serine Repeat (SR) domain; CNC-bZIP domain. Potential sites of post-translational modifications including N-glycosylation [122,123], phosphorylation [124,125,126], O-GlcNAcylation [127,128,129], and N-terminal truncation [93] are also indicated. Figure created with BioRender.com, accessed on 30 July 2023.

## 9. Structural Domains and Post-Translational Modifications of NFE2L1

The human NFE2L1 gene is located on chromosome 17 and spans nine exons. Human NFE2L1 consists of 742 amino acid residues, whereas the mouse protein has 741 residues [130,131] (Figure 2). Other isoforms of NFE2L1 are generated through alternate splicing, including a long human-specific variant named TCF11 that consists of 772 amino acids. A shorter isoform named LCR-F1 can also be generated from an internal Kozak sequence located within the NFE2L1 coding sequence [132]. Like NFE2L1, SKN-1 also exists as multiple isoforms (SKN-1a, b, and c) [133]. The N-terminal domain (NTD) contains protein sequences that target NFE2L1 to the ER. Acidic Domain 1 (AD1), the Asparagine/Serine/Threonine (NST) domain, Acidic Domain 2 (AD2), and the Serine Repeat (SR) domain together constitute the transactivation domains of NFE2L1. AD1 has various subdomains including Neh2L, Neh5L, and Neh4L (the latter domain is only present in TCF11). The C-terminal end of NFE2L1 contains the CNC-bZIP domain that interacts with small Maf proteins, a prerequisite for DNA binding and transcriptional activation. A detailed description of the various NFE2L1 structural domains is provided in recent reviews [92,134].

NFE2L1 is maintained at very low levels in the cell and is continuously cleared by the proteasome both in the cytosol (via ERAD pathway consisting of ubiquitin ligase Hrd1 and VCP) [126] and in the nucleus. Levels of nuclear NFE2L1 are regulated by two F-box proteins, β-TrCP (β-transducin repeat-containing protein) [126] and Fbw7 [135]. F-box proteins are a part of the SCF (Skp1-Cul1-F-box) ubiquitin ligase that function as adapters attracting substrates to the SCF complex, resulting in their ubiquitination and subsequent proteasomal degradation. Mouse NFE2L1 contains a DpSGLpS motif spanning residues 447 to 451 (part of AD2 domain) that resembles the canonical β-TrCP binding sequence, wherein Ser 448 and Ser 451 are potential phosphorylation sites. The site of interaction with Fbw7 is the Cdc4 phosphodegron motif spanning residues 350 to 354 in mouse Nfe2L1, where Ser 350 in the NST domain is phosphorylated by GSK3 (glycogen synthase kinase 3) [124]. Levels of NFE2L1 are also regulated by several deubiquitinating enzymes, including USP7, USP19 (specifically in the ER), and USP15 (in the nucleus), that deubiquitinate NFE2L1 and increase its stability and transcriptional activity [136,137,138].

NFE2L1 undergoes various post-translational modifications that affect its transcriptional activity. One of these is a series of glycosylation/deglycosylation reactions in the NST domain. NFE2L1 is glycosylated in the ER lumen but must undergo a deglycosylation step after being retrotranslocated to the cytosol to form the active transcription factor. In humans, the deglycosylation step is catalyzed by NGLY1, a PNGase that cleaves high-molecular-weight mannose chains from asparagine residues [139]. NFE2L1 glycosylation occurs readily in cells with high glucose levels, whereas deglycosylation is favored under conditions of glucose deprivation [92,122]. These observations suggest that NFE2L1 can function as a glucose sensor, and they imply that the protein’s activation may be regulated by perturbations of glucose metabolism characteristic of NDs such as AD or PD [140,141], in addition to the proteostasis mechanisms highlighted earlier in this review.

NFE2L1 deglycosylation results in the conversion of asparagine to aspartate [139], a change in amino acid sequence that appears to increase the ability of NFE2L1 to induce the transcription of its target genes [142]. Data from a detailed analysis of SKN-1 also suggest that the conversion of asparagine residues to aspartate favors the transcription of genes encoding proteasome subunits [103]. Knockdown of PNG-1 severely affects the survival of worms treated with proteasome inhibitor, suggesting that PNG-1-mediated NFE2L1 deglycosylation and the concomitant conversion of asparagine residues to aspartate are necessary for proteasome subunit expression. NGLY1 deficiency is well characterized in humans and is known to cause neurological symptoms along with motor and developmental deficits [143]. *Drosophila* with decreased levels of NGLY1 exhibit defects in the expression of NFE2L1-regulated genes, including genes encoding proteasome subunits and antioxidant response proteins, suggesting that NFE2L1 dysfunction is a major downstream consequence of NGLY1 deficiency that could account for the neurological symptoms observed in patients with NGLY1 mutations [144]. 

In addition to N-linked glycosylation, NFE2L1 has been reported to undergo O-GlcNAcylation by the enzyme O-linked N-acetylglucosamine transferase (OGT), potentially at the SR domain (amino acids 454–488). O-GlcNAcylation was found to negatively regulate NFE2L1 function by causing increased ubiquitination and degradation of the protein, resulting in decreased stability and transcriptional activity [127]. However, contradictory results were obtained in other studies, where NFE2L1 O-GlcNAcylation (resulting from the binding of NFE2L1 to OGT and the co-factor host cell factor 1 (HCF1) via interactions involving the Neh6L domain) led to increased NFE2L1 stability and transcriptional activity [128,129]. The authors also identified Ser 448 and 451 of mouse NFE2L1 as potential sites for O-GlcNAcylation, and both residues are also phosphorylation sites as described above. Conversion of these residues to alanine via site-directed mutagenesis prevented NFE2L1 O-GlcNAcylation. Phosphorylation and O-GlcNAcylation are competing modifications, suggesting that O-GlcNAcylation of these residues may prevent phosphorylation and subsequent ubiquitination by β-TrCP, ultimately leading to NFE2L1 stabilization. Additional studies focused on the impact of O-GlcNAcylation on NFE2L1 function are necessary to determine the cellular consequences associated with this modification. 

NFE2L1 has also been reported to be phosphorylated at Ser 497 (Neh6L) by casein kinase-2 (CK2), a kinase that negatively regulates NFE2L1 transcriptional activity and causes a decrease in proteasome subunit expression [125]. In contrast to the effects of GSK3 described above, CK2-mediated phosphorylation does not affect the steady-state levels or degradation kinetics of NFE2L1. However, CK2 knockdown was found to cause an increase in the recruitment of NFE2L1 to ARE motifs of target genes, in turn implying that CK2-mediated phosphorylation interferes with NFE2L1-ARE interactions by changing the conformation of the protein.

## 10. Functional Interplay between NFE2L1 and Other Nrf Family Members

The biological functions of the Nrf family of transcription factors have been widely investigated, especially in the case of Nrf2, a key mediator of xenobiotic metabolism and redox homeostasis that plays a major role in the pathophysiology of cancer, neurodegenerative disorders, cardiovascular disease, and metabolic syndromes [145]. NFE2L1 and Nrf3 are ER-bound, whereas Nrf2 is cytosolic. The basal expression of these transcription factors is maintained at a low level through continuous proteasomal degradation. Cellular signals including oxidative stress and proteasome impairment lead to the stabilization of these transcription factors, followed by their translocation into the nucleus where they can activate their target genes. Although NFE2L1 and Nrf2 are ubiquitously expressed in most tissues at all developmental stages, Nrf3 is expressed predominantly in the placenta. Within the central nervous system (CNS), Nrf2 expression has been found to be significantly enriched in non-neuronal populations, whereas NFE2L1 is expressed in all cell types, as revealed by RNA-Seq analysis of wild-type mouse brain [146]. In the same study, low levels of Nrf3 were also detected in the brain, specifically in oligodendrocytes. Collectively, these transcription factors affect the expression of genes involved in a wide range of cellular processes including redox homeostasis, RNA metabolism, lipid metabolism, and protein folding and degradation [147]. 

Although all Nrf family members bind to the ARE [148,149,150], they exhibit slight differences in their preferences for particular ARE motifs and activate distinct (but overlapping) sets of target genes [147]. Some genes are solely regulated by one member of the Nrf family, whereas others are regulated by all three. These differences in target gene profiles could account for differences in the phenotypes observed for different Nrf knockout models. Global knockout of NFE2L1 or LCR-F1 in mice results in embryonic lethality, which may be attributed to increased oxidative stress, deficient erythropoiesis, or lack of globin gene expression [151,152,153]. In contrast, knockout of the Nrf2 or Nrf3 gene does not cause developmental arrest in mice [154,155], although a double NFE2L1/Nrf2 knockout results in embryonic lethality at an earlier stage compared to NFE2L1 deletion alone [156]. These observations suggest that (i) NFE2L1 and Nrf2 have overlapping protective functions, and (ii) Nrf2 may have compensatory protective effects that reduce the severity of the NFE2L1 knockout phenotype. Consistent with this idea, the antioxidant effectors GCLC, GCLM, HO-1, and NQO-1 are expressed at markedly lower levels in fibroblasts obtained from NFE2L1/Nrf2 double-knockout mice compared to NFE2L1 single-knockout cells [156]. 

Studies of NFE2L1- and Nrf2-mediated gene expression in the liver have yielded key insights into the functional interplay between these two transcription factors. Liver-specific deletion of NFE2L1 in mice leads to pathological features similar to those of human nonalcoholic steatohepatitis (NASH) [157]. This phenotype was found to be primarily the result of a reduction in the expression of metallothionein MT-1 and MT-2, but not the antioxidant proteins GCLC, GST, or NQO-1, levels of which were apparently maintained by Nrf2 transcriptional activity. Although both NFE2L1 and Nrf2 were found to bind to the promoters of the MT-1 and NQO-1 genes with comparable affinities, NFE2L1 activated MT-1 transcription to a greater extent than NQO-1, whereas the opposite pattern was observed for Nrf2. These results imply that Nrf2 plays a more significant role in modulating the antioxidant response than NFE2L1. However, a study of fetal livers obtained from NFE2L1-/- mice (prior to embryonic lethality) revealed a significant reduction in glutathione levels and a downregulation of the antioxidant proteins GCLC, GCLM, and HO-1, in addition to MT-1 and MT-2, suggesting that NFE2L1 can also regulate the antioxidant response under certain conditions [153]. Additional analyses of chimeric mice produced by injecting NFE2L1-/- embryonic stem cells into WT blastocysts revealed that NFE2L1-/- stem cells can differentiate into cells of all major organ types except the liver.

Other studies have revealed that in the CNS, Nrf2 expression is significantly lower in neurons compared to astrocytes as a result of epigenetic repression of the Nrf2 promoter, a phenomenon that begins at early stages of neuron development and persists in mature neurons [158]. The absence of Nrf2 in developing neurons creates an oxidative environment that allows for the activation of ROS-sensitive signaling pathways necessary for neuronal maturation. In contrast, NFE2L1 is abundantly expressed in neurons [159] and could thus compensate for the absence of Nrf2 in maintaining redox homeostasis and proteostasis in this cell type. 

NFE2L1 and Nrf2 also have compensatory roles in regulating other pathways, including ferroptosis, a cell-death pathway associated with increased intracellular iron accumulation and lipid peroxidation. A decrease in NFE2L1 or Nrf2 expression in a human epithelial cell line was found to sensitize cells to ferroptosis-inducing agents such as erastin2, although the downstream mediators that produced these effects were distinct for each transcription factor [160]. NFE2L1 downregulation led to a decrease in the expression of glutathione peroxidase 4 (GPX4), an enzyme that prevents lipid peroxidation, whereas a loss of Nrf2 caused a reduction in GCLC expression and a corresponding decrease in glutathione levels. However, NFE2L1 or Nrf2 overexpression rescued erastin2-mediated ferroptosis caused by a loss of Nrf2 or NFE2L1, respectively, suggesting that a certain degree of functional overlap exists between the two transcription factors. 

Overall, these findings suggest that (i) NFE2L1 and Nrf2 regulate the expression of overlapping sets of genes; and (ii) the balance of NFE2L1 and Nrf2 transcriptional activities in relation to particular gene targets (e.g., antioxidant genes) depends on the cellular context and stage of cell differentiation. 

## 11. NFE2L1- and Nrf2-Dependent Regulation of Other Proteostasis Mechanisms

Although NFE2L1 has been studied primarily for its role in regulating the UPS, it has also been shown to be involved in modulating other proteostasis pathways that are impaired in NDs, including the ALP. Flux through the UPS and ALP is coordinated such that an impairment of one pathway causes an upregulation of the other [161]. In support of this idea, Goldberg and colleagues [162] found that exposing SH-SY5Y neuroblastoma cells to a proteasome inhibitor led to an NFE2L1-dependent upregulation of genes involved in the ALP, including p62 and GABARAPL1, in addition to proteasome subunit genes. Another study revealed that the ALP was upregulated together with the proteasome and antioxidant pathways in response to NFE2L1 activation in a model of heart regeneration [163]. Nrf2 has also been shown to bind to the promoter region of the gene encoding the autophagy adapter p62 and stimulate its transcription in human cell lines [164]. Multiple genes involved in the ALP contain putative ARE sites and are activated in an Nrf2-dependent manner in response to oxidative stress [165]. 

Cellular proteostasis is also regulated by a chaperone network (consisting of heat shock proteins such as HSP70 and HSP90) that facilitates proper protein folding and prevents aggregation [166]. The intracellular expression of chaperone proteins is regulated by the Heat Shock Transcription Factor 1 (HSF1), which trimerizes and translocates to the nucleus under conditions of proteotoxic stress [167]. Once in the nucleus, trimeric HSF1 activates target genes encoding molecular chaperones. A significant interplay between proteasome impairment, oxidative stress, and induction of the heat shock response has been reported [168,169]. In one study, it was discovered that the *HSF1* gene could be transcriptionally upregulated by Nrf2 under conditions of oxidative stress in a human fibrosarcoma cell line [170]. Moreover, HEK293T cells overexpressing NFE2L1 were found to have increased levels of RNAs encoding molecular chaperones, including HSPA4, HSPA8, and HSPA9 [171]. However, direct binding of NFE2L1 to the corresponding gene promoters was not verified; thus, further studies are needed to validate molecular chaperones as targets of NFE2L1 transcriptional activity.

## 12. Role of NFE2L1 in Age-Related Phenotypes

Extensive research on mechanisms of aging in *C. elegans* models has led to the identification of several longevity-promoting interventions, including dietary restriction, germline ablation, and the modulation of insulin signaling [172]. These treatments cause alterations in multiple, interdependent signaling and transcriptional networks, including TOR, SKN-1/Nrf, and DAF-16/FOXO. Loss of SKN-1 causes an increase in sensitivity to toxicants such as paraquat and a decrease in lifespan, whereas SKN-1 overexpression increases lifespan [173,174,175]. SKN-1 is also necessary for lifespan extension conferred by inhibition of the TORC1 pathway [115]. Treatment with rapamycin or knockdown of TORC1 signaling effectors induces an increase in the expression of SKN-1 and its gene targets, which is necessary for the induction of protective stress responses and increased lifespan. The role of SKN-1-dependent proteasome regulation in *C. elegans* longevity is not fully understood, in contrast to other SKN-1-mediated protective functions including detoxification and the antioxidant response. Overexpression of pbs-5 (the *C. elegans* ortholog of the PSMB5 proteasome subunit) causes an overall increase in proteasome content and confers an SKN-1-dependent increase in lifespan [176]. However, it is unclear how upregulated proteasome subunit expression is linked to downstream activation of SKN-1, given that SKN-1 is only activated under conditions of proteasome impairment. Lifespan extension and increased proteasome activity are also observed in *C. elegans* strains that lack a germline, namely, *glp-1* mutant worms [174]. Although a knockdown of SKN-1 was found to abolish the increase in lifespan associated with the *glp-1* mutation, it did not affect proteasome activity in these worms. Instead, proteasome content in *glp-1* worms was modulated by a different transcription factor, DAF-16/FOXO. Different transcriptional responses are likely to occur depending on the types of interventions used for lifespan extension, as well as the isoform of SKN-1 that is activated. SKN-1b, a variant only expressed in a set of sensory neurons named ASI neurons, is necessary for the increase in lifespan observed upon dietary restriction, and this effect is at least partially mediated by an increase in mitochondrial respiration [177]. Another major mechanism that regulates aging in *C. elegans* is the insulin signaling pathway. A decrease in insulin signaling causes an extension of the mean worm lifetime, and this phenotype is regulated by SKN-1 in the intestine, along with other transcription factors that act via independent mechanisms [178]. Phosphorylation of SKN-1 by various kinases, including Akt, downstream of the insulin receptor pathway reduces SKN-1 nuclear localization and transcriptional activity. Data from other studies suggest that the three SKN-1 isoforms have different transcriptional targets, with SKN-1a and SKN-1c having more prominent roles in regulating proteasome subunit expression (similar to NFE2L1) and the antioxidant response (similar to Nrf2), respectively [103]. Future studies focused on the role of NFE2L1 in modulating age-related phenotypes in mammalian models are warranted to determine if the SKN-1-dependent responses in *C. elegans* described above are conserved in higher order species.

## 13. Role of NFE2L1 in NDs

Given its role in maintaining proteostasis through the upregulation of UPS subunits, NFE2L1 expression would be expected to have a protective effect in NDs. Although increasing protein clearance through the UPS is considered a viable therapeutic strategy to delay ND progression, it is uncertain whether transcriptional upregulation mediated by NFE2L1 could alleviate neuropathology. NFE2L1 is widely expressed in the brain [159], and mice with a forebrain neuron-specific knockout of the NFE2L1 gene, generated using the Camk2Cre transgenic line, show evidence of neuronal apoptosis and the accumulation of ubiquitinated substrates due to a decrease in proteasome subunit expression and UPS activity [159]. A parallel study involving mice with a CNS-wide deletion of the NFE2L1 gene, generated using the Nestin-Cre transgenic line, revealed a more severe phenotype characterized by early postnatal lethality, an accumulation of ubiquitinated protein aggregates, and increased oxidative stress [179]. Arenas and colleagues [180] reported that midbrain dopaminergic neurons of PD patients had lower levels of nuclear NFE2L1 compared to healthy controls, and that cultured dopaminergic neurons with an NFE2L1 knockdown were sensitized to caspase-3 activation under conditions of oxidative stress. These results suggest that NFE2L1 plays an important role in the antioxidant response in dopaminergic neurons. RNA-Seq analysis of skeletal muscles from mice expressing mutant androgen receptor (AR113Q), a model of spinal and bulbar muscular atrophy (SBMA), revealed a significant reduction in the expression of multiple proteasome subunits [181]. This defect was found to result from a downregulation of the aspartyl protease DDI2, in turn leading to impaired NFE2L1 processing and activation. Bott and colleagues [182] showed that a small-molecule analog of curcumin (ASC-JM17) that stimulates NFE2L1 transcriptional activity reduces the accumulation of another androgen receptor mutant (AR97Q) in the skeletal muscles of AR97Q transgenic mice by upregulating proteasome subunit expression [182]. This compound also had stimulatory effects on Nrf2, causing an upregulation of antioxidant enzymes that in turn led to a decrease in oxidized protein levels. Treatment with ASC-JM17 also resulted in the rescue of AR-induced toxicity in a *Drosophila* model of SBMA. This protective effect was found to be dependent on CncC, the *Drosophila* ortholog of Nrf1/2. Collectively, these data suggest that stimulating NFE2L1 transcriptional activity using small molecules is a viable therapeutic approach.

Although it is well established that proteasome inhibitors can induce NFE2L1 activation, it is unclear whether aggregated proteins can stimulate NFE2L1-mediated signaling by disrupting proteasome function. In a genetic screen designed to identify factors that induce proteasomal subunit expression in *C. elegans*, Lehrbach et al. [175] observed that mutations in *unc-54* triggered SKN-1 activation. UNC-54 encodes a myosin class II heavy chain (MHC-B), and the mutations identified in the screen resulted in misfolding and aggregation of the protein. SKN-1 was also activated in transgenic worms encoding human Aβ, leading to a rescue of the worms’ adult-onset paralysis phenotype, suggesting that aggregated proteins can cause SKN-1 activation. However, contrary to evidence that misfolded proteins can cause proteasome dysfunction (see above), the increase in SKN-1 activation observed in worms expressing mutant UNC-54 or Aβ was not associated with a decrease in proteasome activity. Instead, other factors may have contributed to SKN-1 activation in this study, including a buildup of aggregated proteins in the ER, the site of SKN-1 localization under basal conditions. 

## 14. Conclusions

**Figure 3:**
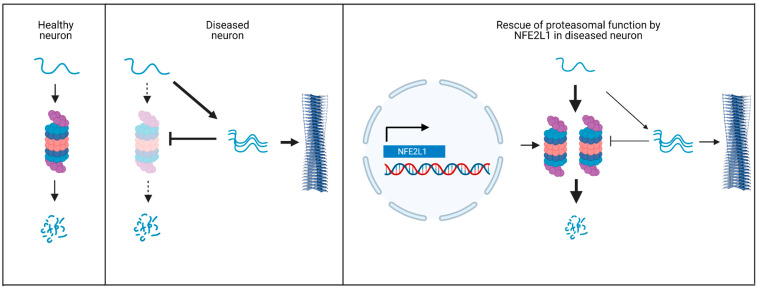
Model illustrating the neuroprotective function of NFE2L1: (**Left**) The UPS plays a major role in the degradation of amyloidogenic proteins such as α-synuclein in healthy neurons. (**Middle**) In neurons from patients with NDs such as PD, α-synuclein undergoes self-assembly to form oligomers and fibrils, a process that is facilitated by age- or aggregate-dependent inhibition of the proteasome. (**Right**) Upregulation of proteasome function via NFE2L1 activation should promote the clearance of monomeric α-synuclein, in turn leading to a decrease in α-synuclein aggregate burden and a slowing of PD progression. Figure created with BioRender.com, accessed on 30 July 2023.

Multiple studies have highlighted the importance of targeting protein clearance mechanisms as a therapeutic approach for NDs. NFE2L1 is a compelling target because of its ability to induce the expression of proteasome subunits, thereby promoting the degradation of amyloidogenic proteins (Figure 3) [183]. As discussed earlier, the exact nature of the neurotoxic species in NDs is still under debate. Importantly, however, our suggested approach of stimulating NFE2L1 function as a neuroprotective strategy for NDs is applicable despite uncertainty about the nature of the toxic species, as lowering the amounts of the monomeric forms of these proteins via enhanced UPS-mediated degradation should lead to a decrease in levels of multiple proteoforms potentially involved in neurodegeneration, including downstream aggregates or variants with aberrant post-translational modifications.

In addition to modulating proteasome subunit gene expression, NFE2L1 regulates various oxidative stress response genes, including genes encoding enzymes necessary for glutathione synthesis. Because Nrf2 is predominantly expressed in non-neuronal cells (e.g., astrocytes), NFE2L1 may play a vital role in maintaining proteostasis and redox homeostasis in place of Nrf2 in neurons. Therefore, strategies to upregulate the transcriptional activity of NFE2L1 in neurons pharmacologically or via gene therapy may be protective against the progression of NDs. Direct modulation of transcription factor activity through small molecules has been considered a challenging task due to the lack of active sites and the presence of long stretches of intrinsically disordered domains [184]. Small-molecule screens have so far only resulted in the identification of proteasome inhibitors as the most potent NFE2L1 activators. However, two non-UPS inhibiting compounds with modest NFE2L1 stimulation have been reported [185]. Future studies aimed at modulating upstream regulatory components in the NFE2L1 pathway or stabilizing the truncated active transcription factor in the nucleus may lead to the development of novel therapies against NDs.

## Data Availability

No new data were created or analyzed in this study. Data sharing is not applicable to this article.
